# Digital Biomarkers for Supporting Transitional Care Decisions: Protocol for a Transnational Feasibility Study

**DOI:** 10.2196/34573

**Published:** 2022-01-19

**Authors:** Despoina Petsani, Sara Ahmed, Vasileia Petronikolou, Eva Kehayia, Mika Alastalo, Teemu Santonen, Beatriz Merino-Barbancho, Gloria Cea, Sofia Segkouli, Thanos G Stavropoulos, Antonis Billis, Michael Doumas, Rosa Almeida, Enikő Nagy, Leen Broeckx, Panagiotis Bamidis, Evdokimos Konstantinidis

**Affiliations:** 1 Medical Physics and Digital Innovation Laboratory School of Medicine Aristotle University of Thessaloniki Thessaloniki Greece; 2 Faculty of Medicine School of Physical & Occupational Therapy McGill University Montreal, QC Canada; 3 Centre de Recherche Interdisciplinaire en Réadaptation Constance-Lethbridge Rehabilitation Center du CIUSSS du Centre-Ouest-de-l’Île-de-Montréal Montreal, QC Canada; 4 Clinical Epidemiology, Centre for Outcomes Research and Evaluation (CORE) McGill University Health Center Research Institute Montreal, QC Canada; 5 Laurea University of Applied Sciences Vantaa Finland; 6 Life Supporting Technologies Universidad Politécnica de Madrid Madrid Spain; 7 Centre for Research & Technology Hellas Information Technologies Institute Thessaloniki Greece; 8 Second Propedeutic Department of Internal Medicine General Hospital “Hippokration” Aristotle University of Thessaloniki Thessaloniki Greece; 9 Fundación INTRAS RDi Projects Department Valladolid Spain; 10 Nagykovácsi Wellbeing Living Lab Nagykovácsi Hungary; 11 Thomas More University of Applied Sciences – LiCalab Antwerp Belgium; 12 European Network of Living Labs Brussels Belgium

**Keywords:** Living Lab, cocreation, transitional care, technology, feasibility study

## Abstract

**Background:**

Virtual Health and Wellbeing Living Lab Infrastructure is a Horizon 2020 project that aims to harmonize Living Lab procedures and facilitate access to European health and well-being research infrastructures. In this context, this study presents a joint research activity that will be conducted within Virtual Health and Wellbeing Living Lab Infrastructure in the transitional care domain to test and validate the harmonized Living Lab procedures and infrastructures. The collection of data from various sources (information and communications technology and clinical and patient-reported outcome measures) demonstrated the capacity to assess risk and support decisions during care transitions, but there is no harmonized way of combining this information.

**Objective:**

This study primarily aims to evaluate the feasibility and benefit of collecting multichannel data across Living Labs on the topic of transitional care and to harmonize data processes and collection. In addition, the authors aim to investigate the collection and use of digital biomarkers and explore initial patterns in the data that demonstrate the potential to predict transition outcomes, such as readmissions and adverse events.

**Methods:**

The current research protocol presents a multicenter, prospective, observational cohort study that will consist of three phases, running consecutively in multiple sites: a cocreation phase, a testing and simulation phase, and a transnational pilot phase. The cocreation phase aims to build a common understanding among different sites, investigate the differences in hospitalization discharge management among countries, and the willingness of different stakeholders to use technological solutions in the transitional care process. The testing and simulation phase aims to explore ways of integrating observation of a patient’s clinical condition, patient involvement, and discharge education in transitional care. The objective of the simulation phase is to evaluate the feasibility and the barriers faced by health care professionals in assessing transition readiness.

**Results:**

The cocreation phase will be completed by April 2022. The testing and simulation phase will begin in September 2022 and will partially overlap with the deployment of the transnational pilot phase that will start in the same month. The data collection of the transnational pilots will be finalized by the end of June 2023. Data processing is expected to be completed by March 2024. The results will consist of guidelines and implementation pathways for large-scale studies and an analysis for identifying initial patterns in the acquired data.

**Conclusions:**

The knowledge acquired through this research will lead to harmonized procedures and data collection for Living Labs that support transitions in care.

**International Registered Report Identifier (IRRID):**

PRR1-10.2196/34573

## Introduction

### Background

#### Transitional Care Study in the Context of the Virtual Health and Wellbeing Living Lab Infrastructure Project

Virtual Health and Wellbeing Living Lab Infrastructure (VITALISE) is a Horizon 2020 project funded by the European Union (under grant 101007990; April 2021 to March 2024), which brings together 19 partners from 11 different countries. The aim of the VITALISE project is the harmonization of services, methods, and procedures of health and well-being Living Labs, enhancing the interaction between multidisciplinary researchers among and beyond the consortium partners. For this purpose, joint research activities (JRAs) will be conducted by the VITALISE consortium. The JRAs will target rehabilitation, transitional care, and everyday life activities, bringing together researchers with diverse expertise across the Living Labs and creating innovation test beds based on harmonized infrastructures, data elements, processes, and research methods. This protocol describes the design of the JRA in the field of transitional care, exploring how information and communications technology (ICT) can act as a facilitator of the creation of big data to predict the risk of adverse events during transitions in care.

#### Transitional Care

Transition periods, most of the time, are related to vulnerable moments in the care of individuals who need to experience frequent transitions between settings, usually from hospital to home or rehabilitation centers and within hospitals from one unit to another. Poorly executed transitions from hospital to home or to rehabilitation centers can potentially result in readmissions, adverse events, patient dissatisfaction, low quality of life, or even death [1]. Approximately half of the patients that are discharged directly to their homes from the hospital are at risk of complications, such as falls, physical deconditioning, aspiration pneumonia, infections, social isolation, and depression because of factors not identified during hospitalization [2-4]. Transitional care is the term used to describe the coordination and continuity of health care to promote safe and timely transitions or *handoffs* between different locations or different levels of care within the same location that is used to minimize risks and improve patient and family experiences [5]. Transitional care includes, but is not limited to, discharge planning, follow-up and care support, patient and family education and supporting self-management, medication management, transfer of information, and shared accountability among providers of patient care [6].

As the provision of transitional care requires multifaceted efforts from the care institutions on both sides of the handoff, the prediction of high-risk patients to provide targeted intervention is of foremost importance [7]. The detection of patients at risk of adverse events or readmissions can guide prevention efforts and prompt proactive care [8] when combined with early treatment of risk factors. Kansagara et al [9] performed a systematic review of risk prediction models using administrative data, either retrospective or using real-time and primary participant data. The recent advances in artificial intelligence and machine learning have introduced computational methods and techniques to improve the prediction of readmissions, avoid the inclusion of *bad data,* and thus predict adverse events with greater precision. Studies have shown that a machine learning algorithm can have better performance for the prediction of readmissions by integrating different factors in the model than commonly used readmission measure scores alone. The potential of deep learning for the prediction of hospital readmissions has also been explored. Wang et al [10] used electronic medical records to predict readmission, whereas Min et al [11] and Xiao et al [12] explored the use of information available in electronic health records and deep learning modeling, which yielded promising results.

ICT can be a facilitator of integrated and coordinated care and can be used to capture biomarkers to develop risk profiles to tailor care to patient’s needs, thereby mitigating future deterioration and optimizing interventions to improve function and participation. ICT can improve transitions in care by (1) standardizing and harmonizing assessment of patients’ function and rehabilitation potential across the continuum of care; (2) exchanging information between interdisciplinary team members and patients or family members, strengthening collaborative care; (3) providing patient and family education and resources to identify services they can access at each point in the continuum of care; and (4) support quality improvement efforts by using data for program evaluation. On the assessment front, ICT tools can be used to provide additional information on a patient’s function in an unobtrusive way that can potentially boost the prediction of readmissions.

Research has shown that data on a patient’s mobility and functional status can predict successful transitions and the risk of adverse events. For example, mobility status is predictive of an increased likelihood that a patient will be discharged to home with better outcomes [13]. Assessment of patients’ functional status may assist in identifying patients at risk for poor outcomes owing to lack of mobility and help describe and quantify patient function [14].

#### Harmonization of Real-life Environments Piloting

Precise predictions of future risk across transitions of care require large data sets to identify the interrelationships among biological, physical, social, and environmental factors. The collection of big data in real-life environment presupposes the exploration of the feasibility of data collection and the development of efficient data acquisition techniques and methodologies [15]. Especially in clinical settings, data may come from many disparate data sources and vary within the local context. Except for data collected from ICT devices, data collected from clinical assessments require special handling on how to be reported, the feasibility of collection from health care professionals, and the differences among health care systems. These challenges emphasize the need for transnational collaboration and harmonization that can on one hand, enhance the exchange of knowledge and technical infrastructure and on the other hand, the exploration of local context views on feasibility and health care systems.

This study helps address the aforementioned challenges for transitional care research through the involvement of Living Lab research infrastructures in 4 countries. Living Labs are defined as ecosystems that enable research activities in realistic environments that drive innovation with multidisciplinary stakeholders [16,17]. They also help adjust research in the local context through the access provided in cross-border real-life research infrastructures and end user populations. In that sense, Living Labs will be exploited with the aim of harmonizing technical infrastructure and outcomes collected by ICT toward supporting transitions of care. Following best practices for implementation science [18], we will develop tools to characterize the context in each Living Lab and to evaluate the barriers and facilitators to implementing ICT and collecting data [19]. The creation of similar sites and the collection of harmonized data sets, even when working with different tools (eg, different types of smartwatches) will support transnational research and collaboration for transitional care around Europe and Canada.

### Objectives

This study will combine data collected using ICT tools, patient-reported outcome measures, and clinical assessments that measure impairments, activity limitations, mobility, and participation of individuals with disabilities or complex chronic conditions to identify digital biomarkers that are related to patient mobility and functional status. This study aims to evaluate the feasibility and perceived benefit of collecting data across Living Labs in 4 countries and to harmonize the data to augment the capacity to perform big data analytics within each local context.

Feasibility across Living Labs include the following:

1. Test and evaluate harmonized infrastructures, data elements, processes, and research methods for transnational collaboration in real-life health care environments for research in transitional care.
2. Evaluate the feasibility to recruit and implement ICT to collect digital biomarkers.
3. Estimate initial patterns of correlation and ability of the data to inform health care transitions.

To inform future scale-up in clinical settings for collecting digital biomarkers, each Living Lab will explore ways of integrating observation of a patient’s clinical condition, patient involvement, and discharge education in transitional care using a simulated hospital environment. Given this is in a simulated environment as compared with a real-world environment, it allows investigators to manipulate how and which ICT is implemented to inform the work in the other Living Labs.

## Methods

### Overview

**Figure 1:**
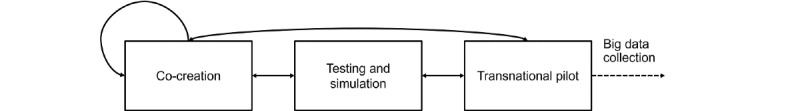
Overview of the information flow for the different phases of the study.

The whole study will consist of three phases that will run consecutively at multiple sites: a cocreation phase, a testing and simulation phase, and a transnational pilot phase. This study is a multicenter, prospective, observational cohort study. The exchange of information among the phases is presented in Figure 1.

The cocreation will be the starting phase that will feed the other 2 but can also create insights that can be further considered in new cocreation sessions, if needed. The transnational pilot could lead to another testing and simulation phase if more data collection needs arise or to cocreation for the exploration of new insights. On the basis of the transnational pilot outcome, a scale-up protocol for big data collection in each Living Lab will be developed.

### Participants and Recruitment Strategies

Different recruitment strategies will be followed for the cocreation, testing and simulation, and transnational pilot phases.

Cocreation will be conducted by each Living Lab; therefore, recruitment will be carried out at each of the stakeholder’s community, including existing collaborations with hospitals and inpatient rehabilitation centers as well as community-dwelling older adults who have previously been hospitalized.

Convenience sampling will be used to recruit nursing students (n=10 per scenario) from the nursing bachelor’s degree program at Laurea University of Applied Sciences for the testing and simulation phase. The simulation scenarios will be integrated into the study units, and a researcher and a lecturer in charge of the study unit will inform the students and ask the voluntary students to participate. Voluntary students will give informed consent to participate.

The recruitment process for the transnational pilot phase will begin during the hospitalization of the patient. The health care professionals who will be in charge of the study at each site will be responsible for recruiting the patients. The patient will be informed about the aims of the study and will be able to ask for any additional information. If they confirm their interest, they will sign the consent form. All participants enrolled in the study will undergo a screening evaluation to determine if they comply with the inclusion and exclusion criteria. The screening evaluation will include two standardized tests: the Clinical Frailty Scale and the Montreal Cognitive Assessment. More specifically, the following are the recruitment sites:

- Thessaloniki Active and Healthy Ageing Living Lab transitions Living Lab in *Hippokration* General Hospital of Thessaloniki targeting a total of 20 participants.
- The McGill–Université de Montréal Biomedical Research and Informatics Living Laboratory for Innovative Advances of New Technologies (BRILLIANT; herein referred to as the BRILLIANT platform) cohort, which will recruit 20 patients and their informal caregivers.
- LifeSpace infrastructure, for which the recruitment of a total of 15 patients will be carried out at hospitals in the Madrid region.

### Study Population and Settings

The cocreation phase will run in different settings across Europe and Canada and will include various stakeholders, such as older adults (>65 years) who have been previously hospitalized, health care professionals, informal and formal caregivers, and family members.

Participants will be included in the transnational pilot study based on the inclusion and exclusion criteria presented in Textbox 1.

**Inclusion and exclusion criteria.**

**Inclusion criteria**

- >65 years, living in the community, who are able to give informed consent
- Admission diagnosis to the hospital that includes one of the following multi-morbidities: stroke or brain injury, rheumatoid arthritis or osteoarthritis, or surgical postoperative patient
- Clinical Frailty Scale <4
- Montreal Cognitive Assessment >25
- Being hospitalized or having recently (within a week) discharged from hospital or inpatient rehabilitation

**Exclusion criteria**

- Severe cognitive disability (eg, not being able to communicate and understand)
- Severe physical disability (eg, tetraplegia)
- Terminal or severe illness with survival prognosis <18 months

### Site and Infrastructure Description

The protocol describes a multisite study that will be performed in four different countries (Greece, Spain, Finland, and Canada). The infrastructures in which the study activities will be performed are described in subsequent sections.

#### Aristotle University of Thessaloniki—Thessaloniki Active and Healthy Ageing Living Lab Health Care Transitions Living Lab (Greece)

The Aristotle University of Thessaloniki (AUTH) Transition Living Lab includes the infrastructure, services, studies, and ICT tools that are used to study and support the transitions of a patient from long-term care facilities such as intensive care unit in hospitals or convalescence after surgery to another long-term care facility such as a rehabilitation center, nursing home, or patient’s house. The AUTH Transitions Living Lab is located in *Hippokration* General Hospital in Thessaloniki, Greece, and consists of a home-like environment equipped with a small kitchen in one room and a bedroom and living room in the other. It is equipped with various ICT tools, including 3D depth sensors (Microsoft Kinect v3.0) for monitoring and analyzing gait patterns and posture, RGB cameras for gesture and activity recognition and emotion analysis, activity trackers, and biosignal monitoring. The collected raw data are analyzed and fused to provide higher level interpretable information to health care professionals. The Living Lab is governed by the Laboratory of Medical Physics and Digital Innovation of AUTH in collaboration with the second Propedeutic Department of Internal Medicine.

#### McGill–Université de Montréal–Centre for Interdisciplinary Research in Rehabilitation of Greater Montreal (Canada)

Canadian hospital centers include infrastructure as part of the Centre for Interdisciplinary Research in Rehabilitation of Greater Montreal–BRILLIANT Community Mobility Rehabilitation. In the Centre for Interdisciplinary Research in Rehabilitation of Greater Montreal–BRILLIANT hospitals and rehabilitation sites, biomedical and health ICT technologies are being evaluated in health care and community settings that are part of the Living Lab network. The Living Lab infrastructure includes a laboratory space of approximately 15,000 sq ft that houses virtual reality research environments, motion caption cameras, haptic devices, wearable sensors, serious game environments, and training simulators. It also includes testing and student spaces, meeting rooms with break out spaces, integrated within the clinical settings. Community Living Labs benefit from meeting and testing rooms, debriefing spaces, and storage for equipment. The Living Labs will also benefit from shared personnel, coordinators, engineers, information technology specialists, and research assistants. There is also a dedicated health informatics team to develop digital health solutions. The Canadian Living Labs are located inside real clinical environments (hospitals and rehabilitation centers), which make them unique environments for constant experimentation and testing of the designed solutions. Although the primary link is with rehabilitation care, the space and network of patients, clinicians, and managers participate in studies across various domains such as transitional care, domotics, and acute care.

#### LifeSTech Living Lab (Spain)

LifeSpace (founded and formally known as Smart House Living Lab by LifeSTech) is an environment that presents an ecosystem approach that combines a wide range of knowledge and stakeholders to offer innovative and personalized solutions aimed at promoting improvement in health and services aimed at social well-being. Within this ecosystem, there is a laboratory for the generation of new knowledge and the creation of new innovative products and services. This environment demonstrates that smart living environments can contribute to and have beneficial effects on quality of life in terms of self-perceived quality of life, physical status perception, social engagement in active and healthy aging, and frailty. On the basis of the European Innovation Partnership in Active and Healthy Ageing Triple Win Strategy, a comprehensive evaluation oriented to data management will be created, designed, and promoted for the generation of global evidence around the three main pillars: quality of life, frailty, and training algorithms for early detection.

More specifically, more than 50 sensors and actuators, iterative robots, internet of things (IoT), and smart devices are distributed in the house. This distribution of *ubiquitous* devices is designed to allow the monitoring and testing of ICT applications, which capture data both within the controlled environment and associated users who actively participate and live at home in the city itself to improve the quality of life and health of citizens, in particular offering gait capture that can, for example, analyze gait improvement to quantify a person’s frailty and early detection of worsening trends because of disease progression or lack of performance of pharmacological treatment.

#### Laurea Simulated Hospital (Finland)

Laurea Simulated Hospital (LSH) provides a Living Lab environment for testing simulation scenarios. The testing process will be incorporated in nursing students’ study units, and the students will practice the transition care process in a safe simulated scenario without causing risk for a real patient. LSH has the potential to be used for scientific validation of developed patient care methods, scenarios, and equipment. There are monitoring and control rooms for the instructors, and a separate debriefing room to be used during the scenarios for observation and after the scenarios for discussions. Simulated scenarios can be monitored in real time and video-recorded for debriefing and research purposes.

#### Centre for Research & Technology Hellas–Information Technologies Institute Near-Zero Energy Building Smart Home (Greece)

The Centre for Research & Technology Hellas–Information Technologies Institute near-zero energy building smart house is a rapid prototyping and novel technology demonstration infrastructure resembling a real domestic building where occupants can experience actual living scenarios while exploring various innovative smart IoT-based technologies with provided energy, health, big data, robotics, and artificial intelligence services. As the first smart near-zero energy building in Greece, it combines enhanced construction materials and intelligent ICT solutions to create a future-proof, sustainable and active testing, validating and evaluating of ecosystems. Data collection and assisted living infrastructure for the purposes of transitional care include IoT smart home sensors (eg, environmental, presence in a room, and appliance use), wearable sensors to capture physiological and lifestyle data, portable electroencephalography devices, robotics, digital coaches or avatars, and mobile apps. After data are collected and stored in an interoperable manner, intelligent data analytics, such as sensor fusion, are used to extract features, behaviors, and symptoms for assessment and care.

### Data Collection

The data collected in this study will consist of qualitative data from the cocreation and testing and simulation phases as well as time series and clinician- and patient-reported outcomes from the transnational pilot phase. The data collection process and data sets are described in the following sections.

#### Cocreation Phase

In the cocreation phase, the objective will be to identify the tendencies of health care professionals, informal caregivers, and patients to use technological tools for collecting information to support their decisions on the transitional process. Possible questions that can be addressed during cocreation sessions are as follows:

1. What are the most important insights or information that a health care professional. informal caregiver, or patient should have about patients as they transition from one care setting to another (focus on hospital to home or hospital to rehab)?
2. Explore the most important expected outcomes of transitional care for a health care professional, informal caregiver, or patient.
3. Explore the experiences that a health care professional, informal caregiver, or patient has from previous transitions in care.
4. What are the needs or desires of health care professional, informal caregiver, or patient as they prepare for a transition and during the transition phase?
5. How can we deliver information to a health care professional, informal caregiver, or patient in an efficient way?

On the basis of the outcomes of the cocreation phase, we will create the first version of the template for reporting the collected data on the simulation and testing and transnational pilot phase. This phase will be used as a first step in defining the local context in the current clinical assessment and to evaluate the required effort from professionals to collect the harmonized data set.

#### Testing and Simulation Phase

##### Overview

Simulation-based research in the health care context can provide opportunities to investigate complex or rarely occurring phenomena, which would be challenging to capture in authentic clinical situations. Furthermore, simulation-based research is a safe approach, especially when vulnerable patient or client groups are involved. It also allows the triangulation of research data and enables patient-public involvement in research design and planning [20]. In the context of this study, simulation-based research will be used at the LSH among trainees and health professionals to explore ways of integrating observation of a patient’s clinical condition, patient involvement, and discharge education in transitional care. The objective of the simulation phase is to evaluate the feasibility and the barriers faced by a health care professional, especially registered nurses (RNs), to assess transition readiness and investigate possible suggestions and directions for support systems. The participants in the testing and simulation phase will evaluate the suggested small-scale pilot, and changes might be applied based on the results.

Simulation scenarios focusing on transitional nursing care will be cocreated with nursing instructors and experts by experience. The scenarios will address (1) discharging a patient from a hospital ward to home and (2) transferring a patient from the emergency department to a hospital ward. The basic structure of the simulation scenario is described in the *Discharging a Patient From a Hospital Ward to Home* section; however, the details will be defined later based on cocreation.

##### Discharging a Patient From a Hospital Ward to Home

Once a physician has decided to discharge the patient and has delivered basic medical information concerning the operation and possible follow-up, an RN checks that discharge criteria are met using Airway, Breathing, Circulation, Disability, Exposure approach. The RN also administers the test battery that has been agreed upon for the small-scale pilot. The RN provides discharge education and instructions about the tasks that they need to perform at home. The RN will also be responsible for assisting the patient in properly placing the wearable devices and ensuring that the sensors work correctly.

##### Transferring a Patient From the Emergency Department to a Hospital Ward

The emergency department physician made the decision to transfer the patient to a hospital ward, where care will continue according to the physician’s orders. The RN taking care of the patient is responsible for transferring the patient to the hospital ward and making appropriate preparations [21], which is to inform the patient, perform patient assessment using the Airway, Breathing, Circulation, Disability, Exposure approach, and check all the documentation and the physician’s orders. An RN in the emergency department gives an oral handover following the Identify, Situation, Background, Assessment, and Recommendation structure to a ward nurse over phone. Identify, Situation, Background, Assessment, and Recommendation is a recommended systematic structure for handovers. It improves information exchange in transition care and promotes desirable patient outcomes and safety [22]. The ward nurse will also test the 3D cameras and app that is placed in the ward to monitor the patient and report on its usability and usefulness.

In both scenarios, a group of nursing students (n=10 per scenario) will play the roles of the patient and RN and act as observers. They will be trained for the roles (especially the patient) before the simulation. A simulation instructor, a researcher, and an expert with knowledge of the specific process will be observing the scenario.

After the simulation scenarios, a debriefing discussion with the student group will take place in the form of a group interview. Interview data will be analyzed using inductive content analysis. Students’ feedback about the simulation scenarios in the form of structured and open feedback will also be gathered. Cocreation workshops will be organized after each simulation session. The aim of the cocreation phase is to develop both the simulation scenarios and the transition care process further. The participants of the simulation scenarios will facilitate the creation of new solutions and provide new insights through an iterative process.

#### Transnational Pilot Phase

**Figure 2:**
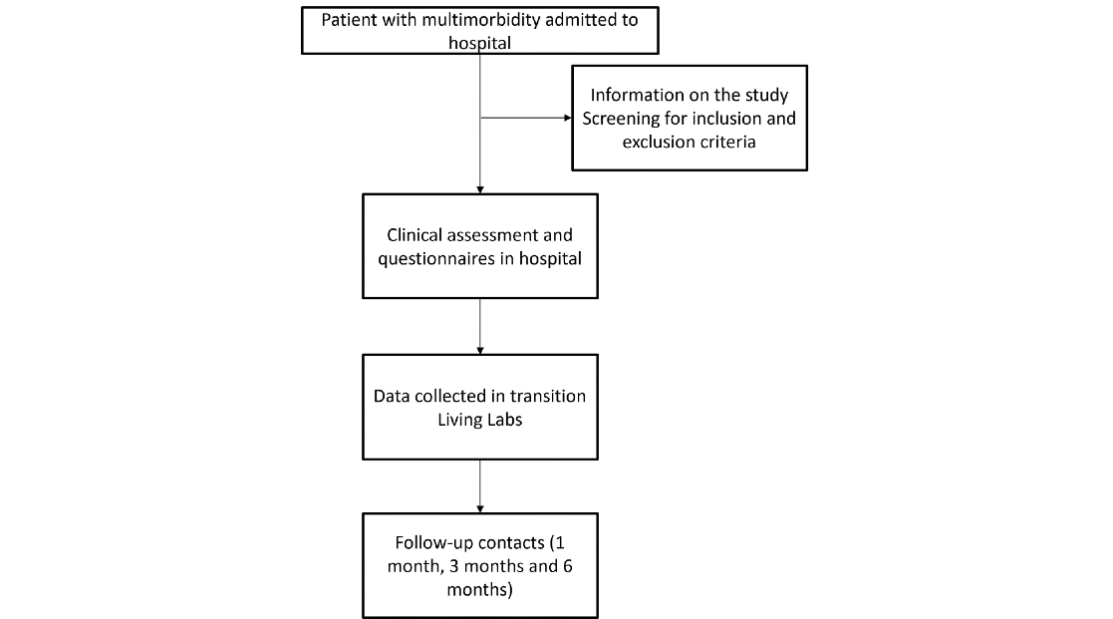
Transnational pilot stages.

The transnational pilot phase will involve the collection of longitudinal multiple time series and clinical data to conduct predictive analytics. The BRILLIANT platform [23] will be used as a reference point for the creation of predictive analytics and the future visualization of outcomes in the included rehabilitation information system (clinicians and patient or caregiver interface). An overview of the transnational pilot activities that will be performed is presented in Figure 2.

The data collection will take place in 3 different time frames, inside the hospital settings during hospitalization, during hospital discharge within transition Living Labs, and within 1 week after discharge. Table 1 summarizes the timing of the data collection.

During their hospitalization in McGill and AUTH clinical sites, a 3D depth sensor camera will be installed in the patient’s ward to monitor the patient’s movements in the bed. The smartwatches that are provided in the recruitment phase will monitor the patient’s oxygen level, pulse rate, heart rate, sleep (heart rate variability details, daily oxygen saturation, and respiratory rate summary), stress (stress score and mindfulness), and blood glucose on a daily basis.

Before leaving the clinical settings (AUTH and McGill) or no more than 1 week after leaving the hospital (LifeSTech and CERTH), the patients will be asked to provide specific demographic features and prehospitalization history. Hospital charts will be used to identify the location of discharge after hospital and health professional–defined care plans. In addition, the patients will enter the transitions Living Labs in which they will remain for a few hours to perform specific measurements. To gather these data in an ecologically valid way, the participants will enter the transitions Living Lab infrastructure for 1 hour and will be asked to perform a morning routine that will include the patient rising from the bed, preparing breakfast, eating breakfast, spending some time watching television, reading a newspaper article or a small section of a book (they will be asked to read it out loud to capture linguistic features), and call a relative or a nurse on the phone. During these activities, the aforementioned sensors will perform measurements unobtrusively.

After discharge, the patient will have three follow-up contacts (1 month, 3 months, and 6 months). In these follow-ups, we will identify the following:

- The occurrence of readmissions within that period
- The occurrence of any adverse events
- The optimal care pathways that the patient was following
- Capture intervention that the patient has done (eg, physiotherapy)—type and intensity
- Participation and social inclusion
- Completion of questionnaires on symptoms, activity limitations, and participation in the BRILLIANT transitions of care platform

**Table 1:** Data collection.

Domain	Measurement tool	Hospital	Hospital discharge and Living Lab	After discharge
Demographic features	Questionnaire filled by a health care professional including gender, date of birth, level of education, employment status, income, living arrangements, and hospital insurance status		✓	
Patients’ health record	Filled by the health care professional		✓	
Prehospitalization history	Including any previous hospitalization, period of hospitalization, and reason for hospitalization		✓	
Health status	EQ-5D-3L^a^	✓		
Health-related quality of life (physical, social, and emotional health)	SF-12^b^ or PROMIS 29^c^ (mapping tables between both exist)	✓		
Depression	PHQ-9^d^	✓		
Functional status	Lawton Instrumental Activities of Daily Living scale	✓		
Risk assessment	BRASS^e^	✓		
Physical activity	IPAQ^f^ or AM-PAC^g^-Inpatient Basic Mobility Short Form Information	✓		
Speech features or linguistic analysis	Speech language pathology assessment	✓	✓	
Cognitive status	MoCA^h^	✓		
Quality of mobility or quality of walking and body posture measures	3D depth sensor cameras (Mentorage). Mentorage device can capture the person’s skeleton		✓	
Gait features including steps, velocity, average distance	Smartwatch sensor		✓	✓
Biosignal measurements including heart rate and blood pressure	Smartwatch sensor	✓	✓	✓
Temperature	Thermometer	✓	✓	
Body weight and composition	Smart scale	✓	✓	
Direct time of treatment	Hospital system or manual measurement from health care professionals	✓		
Hospitalization measures	Number of procedures performed during hospital stay; number of hospital stays with ≥5 days; number of hospital admissions during the previous year; length of stay in hospital (days); and number of emergency department visits within 6 months	✓		

^a^EQ-5D-3L: EuroQol five-dimensional questionnaire.

^b^SF-12: 12-item Short Form Survey.

^c^PROMIS 29: Patient-Reported Outcomes Measurement Information System-29.

^d^PHQ-9: Patient Health Questionnaire.

^e^BRASS: Blaylock Risk Assessment Screening Score.^f^IPAQ: International Physical Activity Questionnaire.

^g^AM-PAC: Activity Measure for Post-Acute Care.

^h^MOCA: Montreal Cognitive Assessment.

### Ethics and Data Management Considerations

Each partner institution involved in this study will submit an institutional review board application or ethical committee application according to the respective national regulations at the latest in December 2021. Informed consent will be obtained from all participants before data collection. The collected data will be exchanged among interested parties pseudonymized to perform a joint analysis. The exchanged data sets will be minimized, and partners will share the minimum amount of data needed to prevent potential risks. An access control list for user and data authentication will be created by each party, and a person responsible is already identified to keep the stored information safe.

### Outcome Measures and Analysis

As the primary objective of this study is to evaluate the feasibility of collecting digital biomarkers within a real-life environment and Living Lab premises for transitional care, the outcomes will focus on the consensus on the activities and collected data. The captured data will be accompanied by feasibility parameters across Living Labs, especially the number of recruits, time to complete assessments, and percentage of missing data. Qualitative outcomes will also be gathered from health care professionals to understand the effort and obstacles for gathering each measure. Mixed method analysis will be carried out by combining quantitative and qualitative data captured to arrive at a consensus on harmonized outcomes and methods.

As a secondary outcome, the initial data patterns will be explored. Descriptive statistics for sociodemographic and clinical characteristics of participants will be calculated, and statistical analysis will identify the correlation of collected digital biomarkers with baseline clinical assessments. A prediction algorithm using machine learning techniques will be created to explore the usability of information by health care professionals. The outcomes will feed and drive the big data collection protocol that will follow.

## Results

In this study, each Living Lab governed by a different entity, will submit an ethical committee application according to the respective national regulations at the latest in December 2021. Recruitment for the cocreation sessions will be completed by February 2022, and the cocreation phase will run until April 2022. The testing and simulation phase will begin in September 2022 and will partially overlap with the deployment of the transnational pilot phase that will start in the same month. The data collection of the transnational pilots will be finalized by the end of June 2023. Data processing is expected to be completed by March 2024. The results will consist of guidelines and implementation pathways for large-scale studies and an analysis for identifying initial patterns in the acquired data.

## Discussion

### Study Significance and Future Research

This JRA protocol is 1 of the 3 JRAs that will be conducted during the Horizon 2020 project *VITALISE*, aiming at three different domains of health and well-being research: rehabilitation, transitional care, and everyday living environments. The main scope of this action is to create transnational collaboration opportunities, facilitating access to Living Lab research infrastructures for all European and international researchers. This specific JRA concerns the field of transitional care and addresses the issue of collecting information across countries that can guide and inform transitions of care. In particular, the described design focuses on the feasibility of using ICT tools for the creation of digital biomarkers and how they can be combined with data collected in clinical settings.

This study investigates the collection, integration, and combination of clinical and patient-reported outcome measures with mobility and functional status automatically collected using ICT tools in real-world clinical settings. Digital biomarker, as a complement, enables continuous monitoring that can be collected remotely in real-life, ecologically valid environment [24]. A seamless assessment of the patient’s health status combined with an investigation of the psychosocial factors and needs, experience, and desires of the different parties can create a multidimensional digital phenotyping that may play a role in improving our knowledge and response for successful transitions [25].

This study will act as a precursor for a large-scale study for the collection of big data on transitional care. The harmonization of procedures for data collection and the identification of obstacles will enable different parties to investigate the prerequisites for scaling-up. Multidisciplinary collaboration, including clinicians, engineers, data scientists, and informal caregivers, along with promising progress in big data collection and analytics, could further help to solve this complex puzzle of extracting meaningful indicators of transition care outcomes. A feasibility study is a crucial step that can help different actors understand the relative strengths and weaknesses of the proposed approach and plan accordingly a solution that can work effectively in real-world clinical settings [26]. The collection and validation of digital biomarkers should not be addressed as a one-time process but rather a longitudinal collection that incorporates adaptations and modifications [27].

With the collection of large-scale data, researchers will be able to identify digital biomarkers associated with mobility and functional status of the patient, which can help predict reduction in length of stay, readmissions, adverse events, and care pathways. Furthermore, the aim will be to evaluate the combination of information from various domains, namely impairments, activity limitations, mobility, and participation collected using ICT for the prediction of transition outcomes. That way, it can act as a facilitator to the execution of better and more effective transitions, with less readmissions and adverse events. The future direction is the development of a pragmatic clinical trial.

On a higher level, this study will allow the involved Living Labs to exchange knowledge and harmonize the technical infrastructure in a broader effort to harmonize the methods, services, and tools used across Living Lab initiatives in the domain of transitional care in real-life health care settings.

### Conclusions

This study presents the design and implementation steps of a JRA that will be performed within the VITALISE project. The knowledge acquired through this research will lead to harmonized procedures and data collection for Living Labs that support transitions in care. In addition, this research contributes to the increase in capacity to perform big data analytics while accounting for each local context and across Living Labs.

## Abbreviations

AUTH: Aristotle University of Thessaloniki
BRILLIANT: Biomedical Research and Informatics Living Laboratory for Innovative Advances of New Technologies
ICT: information and communications technology
IoT: internet of things
JRA: joint research activity
LSH: Laurea Simulated Hospital
RN: registered nurse
VITALISE: Virtual Health and Wellbeing Living Lab Infrastructure
